# Homoarginine Associates with Carotid Intima-Media Thickness and Atrial Fibrillation and Predicts Adverse Events after Stroke

**DOI:** 10.3390/life13071590

**Published:** 2023-07-20

**Authors:** Laura Schwieren, Märit Jensen, Robert Schulz, Susanne Lezius, Elena Laxy, Magalie Milatz, Götz Thomalla, Rainer Böger, Christian Gerloff, Tim Magnus, Edzard Schwedhelm, Chi-un Choe

**Affiliations:** 1 Department of Neurology, University Medical Centre Hamburg-Eppendorf, 20246 Hamburg, Germany; 2 Institute of Clinical Pharmacology and Toxicology, University Medical Centre Hamburg-Eppendorf, 20246 Hamburg, Germany; 3 Institute of Medical Biometry and Epidemiology, University Medical Centre Hamburg-Eppendorf, 20246 Hamburg, Germany; 4 German Centre for Cardiovascular Research (DZHK e.V.) Partner Site Hamburg/Kiel/Lübeck, 20246 Hamburg, Germany

**Keywords:** homoarginine, acute stroke, transient ischemic attack, carotid intima-media thickness, atrial fibrillation

## Abstract

Homoarginine is associated with cardio- and cerebrovascular morbidity and mortality. However, the underlying pathomechanisms remain elusive. Here, we evaluated the association of homoarginine with adverse events (i.e., death, stroke, and myocardial infarction) and carotid intima-media thickness (cIMT) in stroke patients. In the prospective bioMARKers in STROKE (MARK-STROKE) cohort, patients with acute ischemic stroke or transient ischemic attack (TIA) were enrolled. Plasma homoarginine concentrations were analyzed and associated with clinical phenotypes in cross-sectional (374 patients) and prospective (273 patients) analyses. Adjustments for possible confounders were evaluated. A two-fold increase in homoarginine was inversely associated with the National Institutes of Health Stroke Scale (NIHSS) score at admission, cIMT, and prevalent atrial fibrillation (mean factor −0.68 [95% confidence interval (CI): −1.30, −0.07], −0.14 [95% CI: −0.22, −0.05]; and odds ratio 0.57 [95% CI: 0.33, 0.96], respectively). During the follow-up (median 284 [25th, 75th percentile: 198, 431] days), individuals with homoarginine levels in the highest tertile had fewer incident events compared with patients in the lowest homoarginine tertile independent of traditional risk factors (hazard ratio 0.22 [95% CI: 0.08, 0.63]). A lower prevalence of atrial fibrillation and a reduced cIMT pinpointed potential underlying pathomechanisms.

## 1. Introduction

Globally, stroke is the second leading cause of death, accounting for 11.6% of all deaths, and the third leading cause of disability and premature death, accounting for 5.7% of all disability-adjusted life years lost, with atherosclerosis being one common underlying pathology [1]. Improved prevention programs and acute therapy interventions cause a decline in age-standardized incidence, prevalence, and mortality rates. However, the absolute burden of disease has steadily increased from 1990 to 2019 due to aging populations, and morbidity and mortality rates are expected to further increase together with enormous healthcare costs [1]. Based on these epidemiological data, the urgent need for improvement both primary preventive strategies and therapeutic options, can be derived.

While pharmacological treatment of risk factors like hypertension and abnormal lipid levels have substantially lowered the mortality risk, there is still a medical need to improve morbidity and to reduce adverse events; e.g., ischemic stroke and myocardial infarction. Carotid artery intimal-media thickness (cIMT) is one measure of subclinical atherosclerotic burden, with higher values being associated with an adverse cerebrovascular and cardiovascular prognosis [2]. Consequently, cIMT has also been proposed as a surrogate marker for therapeutic interventions directed at lowering atherosclerotic burden [3]. In preliminary work, we and others identified new therapeutic targets to improve the outcome of cerebrovascular and cardiovascular disease; for example, homoarginine [4,5].

Homoarginine is a nonproteinogenic amino acid that differs from arginine by one methylene group [5]. It was first isolated from the seeds of the red pea (*lathyrus cicera*) in 1962 by the English botanist and chemist Ernest Arthur Bell [6]. It is supplied via foods such as red peas, grass peas (*lathyrus sativa*), or lentils (*lens culinaris*) [7]. In the Central European diet, red peas, grass peas, and lentils play a minor role, while these legumes are still used in Southern European (Portugal, Spain, and Italy) cuisine as well as in East Africa (e.g., Ethiopia) and Central and South Asia (India, Bangladesh, Kashmir, and Nepal) [8]. A study that examined the differentiation of exogenous and endogenous protein in the chyme of miniature pigs demonstrated that homoarginine was almost completely absorbed in the jejunum and ileum [9]. The absorption of homoarginine in the human gastrointestinal tract is not well studied yet. 

Endogenously, homoarginine is produced in kidneys, liver, and pancreas by the mitochondrial enzyme arginine:glycine amidinotransferase (AGAT). Yet, the alimental intake of homoarginine seems to play a minor role, and it remains to be examined how much homoarginine is derived from food versus enzymatic production [5]. AGAT catalyzes the synthesis of L-homoarginine from L-arginine and L-lysine, while arginase can at least in principle hydrolyze L-homoarginine to L-lysine and urea [10]. However, high L-homoarginine concentrations inhibit arginase, which consecutively leads to increasing arginine and nitric oxide (NO) concentrations, improving vascular homeostasis [11]. Additionally, L-homoarginine serves as a direct substrate for endothelial NO synthase (eNOS) [12]. Furthermore, L-homoarginine is a specific uncompetitive inhibitor of tissue-nonspecific alkaline phosphatase (TNAP). TNAP is expressed in many tissues, including vascular endothelial cells. Epidemiological studies have indicated that elevated TNAP concentrations in blood are an independent predictor of overall mortality in patients with stroke and other cardiovascular diseases [13]. The enzyme alanine:glyoxylate aminotransferase 2 metabolizes L-homoarginine to 6-guanidino-2-oxocaproic acid, whereas some other enzymes of the urea cycle could be involved in the metabolism as well [14]. In addition to enzymatic clearance, homoarginine is also excreted into the urine [5]. However, the extent of hepatic and renal excretion seems to be moderate, and homoarginine can be determined in plasma and serum at median concentrations of 1.77 [25th, 75th percentile: 1.38, 2.26] μmol/L and 2.56 [1.99, 3.32] µmol/L, respectively, in women and 2.01 [1.61, 2.56] and 2.72 [2.20, 3.33] µmol/L, respectively, in men [15,16]. Of note, during pregnancy mean serum homoarginine concentrations increase up to 5.3 ± 1.5 µmol/L in the third trimester [17].

Previously, we associated homoarginine in the population-based Dallas Heart Study inversely with subclinical atherosclerosis; i.e., aortic wall thickness, and homoarginine was found to predict cardiovascular morbidity and mortality [18]. Similarly, a cohort study postulated a strong relationship between arginine and the NO pathway with aortic atherosclerosis but not with aortic distensibility, suggesting different mechanisms in aortic wall remodeling [19]. In cerebrovascular patients, the ratios of homoarginine over asymmetric dimethylarginine and of homoarginine over symmetric dimethylarginine can discriminate stroke etiologies, predict internal carotid artery stenosis, and estimate the risk of atrial fibrillation [20]. In addition to atherosclerotic phenotypes, cardio-embolic etiologies, especially those due to atrial fibrillation, are a main and independent cause of ischemic stroke. Previous studies demonstrated that homoarginine is also inversely associated with corrected QT interval duration and seems to predict prevalent atrial fibrillation [21] as well as advanced atrial fibrillation progression phenotypes [22]. In 3331 participants of the Framingham Offspring Study, lower plasma homoarginine concentrations were associated with higher all-cause mortality during a median follow-up of 18 years [23]. Recently, a meta-analysis of 13 studies that included 11,964 participants confirmed the inverse association of homoarginine and all-cause mortality. Of note, low levels of homoarginine were strongly associated with severe strokes and also revealed a trend toward increased risk for stroke [24]. We therefore examined the blood of patients with acute stroke or transient ischemic attack (TIA) and analyzed cross-sectional and prospective associations of plasma homoarginine with medical conditions as a risk factor for stroke (prevalent atrial fibrillation), cIMT as a parameter for the extant prevalent atherosclerosis, and incident events during a follow-up period in an independent stroke cohort.

## 2. Patients and Methods

### 2.1. Study Design, Ethical Approval, and Patient Consent

The bioMARKers in STROKE (MARK-STROKE) cohort is a prospective observational single-center study at the University Medical Center Hamburg-Eppendorf that recruited 413 patients from the stroke unit at the Department of Neurology from November 2017 until August 2019 [25]. Firstly, every stroke unit patient assumed to have had an acute stroke or TIA was eligible for this study and was informed about this study. For strongly affected patients, a legal representative was allowed to give informed written consent. Exclusion criteria were acute or chronic impaired renal function and serious substance abuse. After exclusion of patients without a final diagnosis of an acute ischemic stroke or a TIA, 374 patients were enrolled.

The inclusion criteria were age >18 years, diagnosis of stroke or TIA at discharge, and written informed consent. From December 2019 until March 2020, all patients were followed up by phone, mail, or e-mail. A total of 273 patients or relatives could be reached, and adverse events (i.e., death, nonfatal myocardial infarction and stroke) were recorded. Information on adverse events was either obtained from patient records, reported by the patients themselves, or given by relatives of the deceased.

The study was conducted according to the guidelines of the Declaration of Helsinki and approved by the Ethics Committee of the Hamburg Board of Physicians (PV4715). Informed written consent was obtained from all subjects involved in the study.

### 2.2. Clinical Assessment

Neurological deficits were assessed via the National Institute of Health Stroke Scale (NIHSS), and the cIMT was calculated using ultrasound measurements at admission. These assessments were conducted by experienced residents from the Department of Neurology. All data, including demographic parameters, past medical history, comorbidities (arterial hypertension, hyperlipidaemia, diabetes mellitus, atrial fibrillation, prior stroke, and prior myocardial infarction), laboratory parameters (glycated hemoglobin A1c (HbA1c), glomerular filtration rate (GFR), low-density lipoprotein cholesterol (LDL-C), high-density lipoprotein-cholesterol (HDL-C), and triglycerides) and cIMT measured via ultrasound were collected as part of the standard stroke unit routine and obtained from the medical records of each patient, as previously described [20,25]. Medication on admission was evaluated for blood-thinning drugs, including acetylsalicylic acid, adenosine diphosphate receptor antagonists, and anticoagulants. Lipid-lowering drugs comprised statins, fibrates, and ezetimibe. The antihypertensive medication consisted of diuretics, angiotensin-converting enzyme inhibitors, angiotensin II receptor blockers, beta adrenoceptor blockers, and calcium channel blockers. The GFR was calculated using the abbreviated MDRD equation: 186 × (creatinine/88.4) − 1.154 × (age) − 0.203 × (0.742 if female) × (1.210 if Black). For sonography of the right and left common carotid arteries, a high-resolution B-mode and duplex ultrasound using a GE Logiq7 system with a 7.5 MHz linear-array transducer (GE Healthcare, Solingen, Germany) was applied. In brief, cIMT was measured at the far wall, and the distance from the leading edge of the first echogenic line to the leading edge of the second echogenic line with 10 mm proximal to the reference point at its thickest point in a region free of plaques was calculated. The greatest cIMT measured in the right and left common carotid artery was used to define the individual cIMT. All carotid ultrasound studies were performed by two investigators, and offline analyses of all frozen ultrasound images were performed by the same experienced reader, who was blinded to clinical data.

### 2.3. Liquid Chromatography–Tandem Mass Spectrometric Measurement of Homoarginine

As part of the admission routine, venous blood samples were taken from every patient participating in this study. In addition to the four standard laboratory monovettes (serum-gel, coagulation, EDTA K, and lithium–heparin; Sarstedt, Nümbrecht, Germany), one further monovette (EDTA K, 7.5 mL; Sarstedt) was taken for this study. The monovettes were centrifuged at 4 °C at 1000 rpm for 20 min. About 3 mL of the supernatant plasma from the monovettes was pipetted into two Eppendorf tubes and frozen at −80 °C.

The laboratory measurements were obtained from these blood samples collected at baseline and processed as previously described with minor modifications [26,27]. In brief, 25 μL of EDTA plasma, calibrator, or quality control sample was subjected to protein precipitation with 100 μL of methanol containing ^13^C_7_^15^N_4_-L-homoarginine (2.5 µmol/L of ^13^C_7_^15^N_4_-homoarginine; Cambridge Isotope Laboratories Inc., Andover, MA, USA) as the internal standard. Residues were derivatized to their butyl ester derivatives, and reconstituted samples were separated on an AQUITY UPLC BEH C_8_ 1.7 µm (2.1 × 75 mm) column (Agilent Technologies, Santa Clara, CA, USA) using an elution gradient of the two mobile phases: (A) 0.1% formic acid in water and (B) 0.1% formic acid in acetonitrile at a flow rate of 0.4 mL/min for 3.2 min. The mean retention time of homoarginine was 1.055 ± 0.014 min (n = 10). Quantification was performed with a Xevo Triple Quadrupole mass spectrometer (Agilent Technologies) with positive electrospray ionization in multiple-reaction mode (MRM). The MRM transitions monitored were for homoarginine *m*/*z* 245.16 > 211.13 @ 16 eV and *m*/*z* 256.20 > 220.17 @ 16 eV for the internal standard ^13^C_7_^15^N_4_-homoarginine. Peak area ratios of the analyte and internal standard were calculated for calibration (four levels; i.e., 0, 2, 5, and 10 µmol/L), quality control (QC-low and -high), and study samples and used for quantification. Quality control samples were accepted below a 15% relative standard deviation (RSD).

### 2.4. Statistical Analyses

Continuous variables are given as the mean ± standard deviation (SD) if normally distributed; otherwise they are given as the median [25th, 75th percentile], and categorical variables are given as numbers (percentage) of participants. Relationships between homoarginine and continuous variables were assessed via Spearman correlation or linear regression analyses on log-transformed variables (exponentiated beta coefficient (=mean factor) and 95% confidence interval (CI) are reported). Statistical comparisons of tertiles were made using one-way ANOVA, Kruskal–Wallis, or Chi^2^ tests as appropriate. For associations with NIHSS and cIMT, we calculated beta coefficients. For statistical comparisons of AF groups, we used logistic regression analyses with odds ratios (ORs), and the corresponding 95% CIs were reported. Univariate associations between homoarginine and events were illustrated via Kaplan–Meier plots and compared with a Mantel–Cox log-rank test. The independent associations between homoarginine tertiles and the time to events were determined via multivariable Cox regression analyses, with results presented as the hazard ratio (HR) and corresponding 95% CI. We adjusted the beta coefficients, ORs, and HRs by applying different models: unadjusted (model 1); adjusted for age and sex (model 2); and additionally adjusted for hypertension, diabetes, smoking, hypercholesterolemia, and GFR (model 3).

A *p*-value < 0.05 was considered statistically significant. Statistical analysis was performed using IBM SPSS Statistics (version 22, IBM Corp., Armonk, NY, USA) and GraphPad Prism (version 5 for Windows, La Jolla, CA, USA).

Deidentified patient data and related documents such as the study protocol and statistical analysis plan will be shared upon request from any qualified investigator for 3 years after the date of publication.

## 3. Results

### 3.1. Homoarginine Levels and Stroke Risk Factors

The 374 patients in the MARK-STROKE cohort were 67.9 ± 13.0 years old, and 64.7% were male. The mean age of patients decreased from 72.9 ± 11.6 years to 63.3 ± 12.6 years from the lowest to the highest tertile of homoarginine, with homoarginine plasma concentrations below 1 µmol/L and above 1.5 µmol/L, respectively (Table 1). The frequency of male patients was higher in the third and second tertile as compared with the first tertile of homoarginine; i.e., 74.4%, 73,4%, and 46.4%, respectively (Table 1). Across increasing homoarginine tertiles, we observed more smokers and a higher GFR but less atrial fibrillation, lower NIHSS scores, and a decreased cIMT (Table 1). Similarly, homoarginine concentrations were positively correlated with higher triglyceride levels and GFR but inversely correlated with age, NIHSS score at admission, and cIMT (Table 2). In multivariable analyses, only the association between homoarginine concentrations and NIHSS, cIMT, and atrial fibrillation remained significant in adjusted regression models (NIHSS: mean factor −0.68 [95% CI: −1.30, −0.07]; cIMT: −0.14 [95% CI: −0.22, −0.05]; atrial fibrillation: OR 0.57 [95% CI: 0.33, 0.96], model 3 (Table 3).

### 3.2. Homoarginine Levels and Stroke Outcome

**Figure 1:**
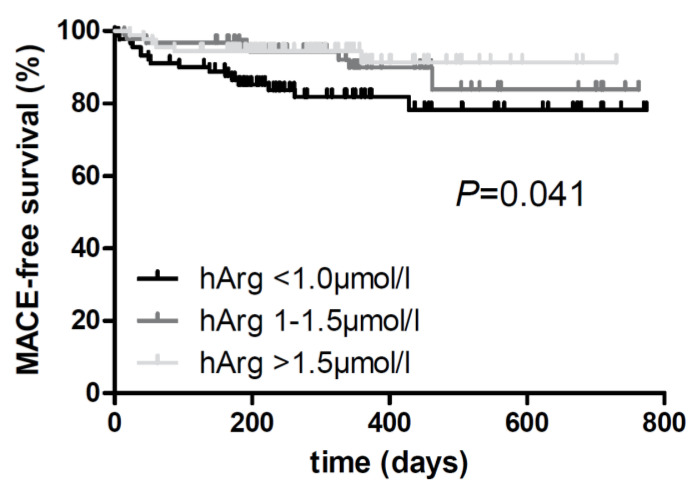
Kaplan–Meier curves for homoarginine tertiles during a median follow-up time of 284 [25th, 75th percentile: 198, 431] days after stroke (lowest tertile: <1.0 µmol/L; second tertile: 1.0–1.5 µmol/L; highest tertile: >1.5 µmol/L). Univariate associations between homoarginine and events were compared using a Mantel–Cox log-rank test (n = 273). Abbreviations: hArg, homoarginine; MACE, major adverse cardiovascular event (i.e., death, nonfatal stroke, and myocardial infarction).

During follow-up (median 284 [25th, 75th percentile: 198, 431] days), we registered 30 major adverse events among 273 stroke patients. A total of 18 patients died during the follow-up period. The causes of death were cardiac, malignant, organ failure, lethal stroke, or of other/unknown etiology. One patient suffered from a nonfatal myocardial infarction; 11 patients had another stroke. In the Kaplan–Meier plots, lower homoarginine levels were associated with incident events across homoarginine tertiles (Figure 1; *p* = 0.041, log-rank test). In the unadjusted and adjusted Cox regression analyses, patients in the highest homoarginine tertile had a significantly lower risk of events compared with the lowest homoarginine tertile (HR 0.22 [95% CI: 0.08, 0.63], model 3 (Table 4)).

## 4. Discussion

Cerebro- and cardiovascular disease are still the leading causes of death worldwide. In preliminary work, we and others identified potential therapeutic targets to prevent or monitor disease and treatment; i.e., homoarginine and its anabolic enzyme AGAT [4,5]. In experimental models of AGAT deficiency, we investigated the functional and mechanistic role of homoarginine to elucidate underlying (patho)mechanisms of atherosclerosis and metabolic disorders [4,28]. In the MARK-STROKE clinical cohort, we now show that a two-fold increase of homoarginine is inversely associated with (1) cIMT and (2) prevalent atrial fibrillation and (3) that individuals with homoarginine levels in the highest tertile had fewer incident events compared with patients in the lowest homoarginine tertile independent of traditional risk factors.

In a large US population-based study with more than 3500 participants, we associated low homoarginine with increased aortic wall thickness; however, in 78 patients with stroke or TIA, homoarginine was neither correlated with aortic stiffness nor with aortic intima-media thickness [18,19]. In line with our previous results, a study on 5-year changes in carotid wall thickness in a South African cohort showed that higher levels of homoarginine played a protective role against vascular injury and delayed progression of carotid wall thickening [29]. We can now provide evidence that the cIMT inversely associates with homoarginine in stroke and TIA patients independently of other risks for atherosclerosis like hypertension, diabetes, hypercholesterolemia, or smoking. Of particular note, not only does the cIMT decrease with increasing tertiles of homoarginine but also the functional decline as measured by NIHSS, which improves with increasing tertiles of homoarginine. This association might depend on the severity of the atherosclerotic burden. In a study of 40 healthy children and adolescents, neither homoarginine nor other arginine derivates correlated with cIMT, suggesting that the inverse association of homoarginine und cIMT might be age- and disease-dependent [30]. Supplementation with homoarginine improves the outcome after acute ischemic stroke and supresses T-cell proliferation and migration in diet-induced atherosclerosis in mice [31]. Moreover, in the second and third trimester of pregnancy, increasing homoarginine was found to be related to improved flow-dilated vasodilation [17]. Taking these results together, homoarginine might be a sharpened sword that ameliorates atherosclerosis by suppressing the immune response and improving vascular function.

Further, we uncovered an association of low homoarginine levels with atrial fibrillation in patients with acute stroke or TIA. Atrial fibrillation causes a detrimental increase in morbidity and mortality and elevates the risk of cardiogenic stroke. Previously, we found that patients with persistent atrial fibrillation at the time of blood sampling had the lowest homoarginine concentrations compared to paroxysmal atrial fibrillation [22]. In contrast, homoarginine levels did not correlate with the occurrence or persistence of atrial fibrillation in a population-based study [32]. Therefore, it seems likely that the association of homoarginine with atrial fibrillation depends on the underlying pathology.

Finally, we showed that low homoarginine is one predictor of incident events after stroke or TIA. In patients after stroke, comprehensive screening tools and risk assessments are needed to prevent further vascular events. For comparison, in patients with acute chest pain, low plasma homoarginine also was a risk marker for major adverse events (death, nonfatal myocardial infarction, and stroke), in particular for those with elevated high-sensitive troponin I [21]. Therefore, low homoarginine levels might be used to identify patients at increased cerebrovascular and cardiovascular risk to improve their aftercare. In the North West province arm of the Prospective Urban and Rural Epidemiology (PURE) South African study, it was examined whether homoarginine was associated with a 10-year risk of all-cause and cardiovascular mortality in a Black South African population. Cardiovascular mortality was defined as death due to cardiac failure, myocardial infarction, or stroke. The participants who survived had higher homoarginine plasma concentrations compared with those who died during the follow-up-period [33]. In another analysis from the PURE South African study, the plasma homoarginine levels of 166 participants who developed arterial hypertension were compared with the plasma homoarginine levels of 166 participants who remained normotensive for a follow-up period of 10 years. A positive association of plasma homoarginine with blood pressure in participants who remained normotensive could be demonstrated, suggesting a protective role of homoarginine for preserving normal blood pressure [34]. These findings also suggest a potential indirect protective effect of high homoarginine for individuals at acute stroke or TIA risk by ameliorating stroke risk factors such as hypertension.

Analyses from the population-based Young Finns Study (n = 2106; 54.6% females, aged 24–39) aimed to evaluate the predictive value of homoarginine in the development of cardiometabolic risk factors (hyperglycaemia, abdominal obesity, and type 2 diabetes) and outcomes like fatal stroke, heart failure, or sudden cardiac death [35]. A Mendelian randomization approach was used to test causal associations. In the 10-year follow-up analysis, homoarginine served as an independent predictor but did not reveal evidence of causal associations between serum homoarginine and cardiometabolic outcomes. Here, elevated concentrations of circulating homoarginine did not seem to change cardiometabolic disease risk [35].

The median homoarginine plasma concentration of our patients in the lowest tertile was 0.77 [0.59, 0.87] µmol/L. Recently, we defined the sex-specific 2.5th and 97.5th percentiles of reference intervals of homoarginine plasma concentrations to be 0.84 and 3.89 μmol/L in women and 0.98 and 4.10 μmol/L in men, respectively [16]. The median homoarginine plasma concentrations of patients in the lowest tertile were barely within the lower 2.5th percentile of the reference intervals and represented the highest risk for major adverse events. Interestingly, homoarginine can be supplemented to healthy humans without harm by applying a once-daily dosing of 125 mg [36]. This dosage was well tolerated without vascular or neurological abnormalities or any other significant side effects [37]. Our findings provide a clear rationale to conduct prospective studies with homoarginine supplementation [38]; e.g., such a study has been initiated in patients with acute ischemic stroke (ClinicalTrials.gov identifier: NCT03692234).

The limitations of these findings were a relatively small sample size and that the study did not include a control group, thus the prevalence of stroke and homoarginine levels in the population were not determined. Also, no records of eating habits were obtained, although the alimental effect of homoarginine ingestion seemed to be small, and foods containing noteworthy amounts of homoarginine are very rare in the standard Central European diet. Furthermore, there was a selection bias because our patients had to be capable of giving informed written consent. For strongly affected patients, a legal representative was allowed to give informed written consent alternatively. This reduced the number of participating patients with severe stroke or a high NIHSS score. In line with this notion, the mean NIHSS scores were relatively low, meaning most patients of our study had minor strokes.

## 5. Conclusions

In conclusion, low homoarginine significantly correlates with advanced arteriopathy, atrial fibrillation, and major adverse events in patients with recent stroke or TIA. Therefore, homoarginine may be a mediator that prevents mortality and morbidity in cerebrovascular patients. However, the evidence for causality is weak, and it remains to be determined if homoarginine is a protective cardiovascular mediator or a clinically useful biomarker of cerebrovascular and cardiovascular risk. Clinical studies that examine the effect of homoarginine supplementation in patients after acute stroke or TIA could provide further insights into possible positive effects of elevated plasma homoarginine levels in stroke pathology.

## Figures and Tables

**Table 1:** Baseline characteristics MARK-STROKE.

Characteristics	1st Tertile (n = 125) <1.0 µmol/L	2nd Tertile (n = 124) 1–1.5 µmol/L	3rd Tertile (n = 125) >1.5 µmol/L	*p*-Value
Demographic parameters				
Age, years	72.9 (11.6)	67.4 (13.1)	63.3 (12.6)	<0.001 ***
Male sex, %	58 (46.4)	91 (73.4)	93 (74.4)	<0.001 ***
Smoking, %	20 (16.0)	42 (33.9)	32 (25.6)	0.004 **
Hypertension, %	91 (72.8)	92 (74.2)	87 (69.6)	0.815
Hyperlipidaemia, %	34 (27.2)	44 (35.5)	43 (34.4)	0.315
Diabetes, %	19 (15.2)	27 (21.8)	17 (13.6)	0.259
Atrial fibrillation, %	37 (29.6)	29 (23.4)	15 (12.0)	0.003 **
Prior myocardial infarct, %	15 (12.0)	11 (8.9)	15 (12.0)	0.660
Prior stroke, %	15 (12.0)	28 (22.6)	19 (15.2)	0.071
BMI, kg/m^2^	26.7 (4.2)	25.8 (4.3)	26.5 (4.9)	0.278
Laboratory parameters				
HbA_1c_, %	5.7 [5.4, 6.0]	5.7 [5.4, 6.2]	5.6 [5.4, 6.0]	0.841
GFR, mL/min	72 [55, 89]	77 [62, 92]	84 [67, 97]	0.001 **
Triglycerides, mg/dL	112 [87, 160]	124 [93, 171]	128 [97, 199]	0.014 *
HDL, mg/dL	49 [40, 60]	49 [40, 61]	46 [38, 56]	0.319
LDL, mg/dL	105 [81, 134]	108 [75, 137]	101 [75, 132]	0.710
Homoarginine, µmol/L	0.77 [0.59, 0.87]	1.22 [1.11, 1.34]	1.94 [1.66, 2.21]	<0.001 ***
Medication				
Blood-thinning, %	105 (84.0)	107 (86.3)	109 (87.2)	0.756
Lipid-lowering, %	81 (64.8)	94 (75.8)	95 (76.0)	0.078
Antihypertensive, %	93 (74.4)	90 (72.6)	85 (68.0)	0.512
Neurological parameters				
NIHSS, points	2 [0, 4]	2 [0, 3]	1 [0, 3]	0.019 *
cIMT, mm	1.4 [1.1, 1.8]	1.2 [1.0, 1.6]	1.2 [1.0, 1.4]	<0.001 *

Continuous data are presented as the mean (SD), n (%), or median [25th, 75th percentile], as appropriate. Categorical variables are given as numbers (percentages) of participants. ANOVA, Kruskal–Wallis or Chi2 tests were used for comparisons between patient’s characteristics categorized by homoarginine tertiles (* *p* < 0.05; ** *p* < 0.01; *** *p* < 0.001). Abbreviations: cIMT, carotid intima-media thickness; GFR, glomerular filtration rate; HbA1c, glycated hemoglobin A1c; HDL, high-density lipoprotein; LDL, low-density lipoprotein; NIHSS, National Institute of Health Stroke Scale.

**Table 2:** Correlation of homoarginine with clinical and laboratory parameters in MARK-STROKE.

	Correlation Coefficient r	FDR-Corrected *p*–Value
Age	−0.312	<0.001 *
BMI	−0.042	0.431
NIHSS at admission	−0.140	0.007 *
GFR	0.225	<0.001 *
Triglycerides	0.159	0.002 *
cIMT	−0.232	<0.001 *

Spearman correlation coefficients with FDR-corrected *p*-values. * Indicates statistical significance after FDR adjustment for 6 tests.

**Table 3:** Linear and logistic regression analysis of homoarginine concentrations with NIHSS, cIMT, and AF.

	Model	NIHSS		cIMT		AF	
		Mean Factor [95% CI]	*p*-Value	Mean Factor [95% CI]	*p*-Value	Odds Ratio [95% CI]	*p*-Value
Homoarginine (per 2-fold increase)	1	−0.77 [−1.35, −0.19]	0.010 *	−0.18 [−0.26, −0.10]	<0.001 ***	0.41 [0.25, 0.67]	<0.001 ***
	2	−0.65 [−1.27, −0.04]	0.038 *	−0.13 [−0.22, −0.05]	0.002 **	0.53 [0.32, 0.89]	0.017 *
	3	−0.68 [−1.30, −0.07]	0.030 *	−0.14 [−0.22, −0.05]	0.002 **	0.57 [0.33, 0.96]	0.036 *

Linear and logistic regression analysis with beta coefficients and odds ratios [95% confidence interval (CI)]. Model 1: unadjusted; model 2: adjusted for age and sex; model 3: additionally adjusted for hypertension, diabetes, hypercholesterolemia, smoking, and GFR (* *p* < 0.05; ** *p* < 0.01; *** *p* < 0.001). Abbreviations: AF, atrial fibrillation; NIHSS, National Institute of Health Stroke Scale; cIMT, carotid intima-media thickness.

**Table 4:** Cox regression analysis of high homoarginine to predict incident events.

	Model	3rd Tertile Versus 1st Tertile	
		Hazard Ratio [95% CI]	*p*-Value
Risk of major adverse events	1	0.37 [0.14, 0.94]	0.036 *
	2	0.24 [0.09, 0.66]	0.006 **
	3	0.22 [0.08, 0.63]	0.005 **

Cox regression analysis with hazard ratios [95% confidence interval (CI)]. Model 1: unadjusted; model 2: adjusted for age and sex; model 3: additionally adjusted for hypertension, diabetes, hypercholesterolemia, smoking, and GFR; n = 273 (* *p* < 0.05; ** *p* < 0.01). Major adverse events were death, nonfatal stroke, and myocardial infarction.

## Data Availability

The data presented in this study are available on request by a qualified investigator for 3 years after the date of publication from the corresponding author.
